# The Impact of Orthodontic Bands on the Marginal Periodontium of Maxillary First Molars: A Retrospective Cross-Sectional Radiographic Analysis

**DOI:** 10.2174/1874210601812010312

**Published:** 2018-04-30

**Authors:** Sabine Teubner, Patrick R. Schmidlin, Giorgio Menghini, Thomas Attin, Stefan Baumgartner

**Affiliations:** 1 Private Practice, Langenthal, Switzerland; 2 Clinic of Preventive Dentistry, Periodontology and Cariology, Center of Dental Medicine, University of Zurich, Zurich, Switzerland; 3 Clinic of Orthodontics and Pediatric Dentistry, Center of Dental Medicine, University of Zurich, Zurich, Switzerland

**Keywords:** Alveolar bone level, Periodontal ligament space, Orthodontic treatment, Molar bands, Marginal periodontium, Radiographic analysis

## Abstract

**Aim:**

Available information on the effect of orthodontic treatment on crestal alveolar bone levels measured in radiographs is contradictory. The aim of this study was to compare the alveolar bone level and periodontal ligament space of banded upper first molars to untreated controls.

**Materials and Methods:**

This retrospective cross-sectional radiographic study investigated alveolar bone levels of upper first molars of an orthodontic test group and an untreated control group of comparable age (15-16.25 years), using existing bitewing radiographs.

Eighty-six individuals were included in each group. Three parameters were measured mesially and distally on both sides of the patient as follows: I) Alveolar Bone Level (ABL): measured as the distance between the cemento-enamel junction and the alveolar crest, II) the Periodontal Ligament Space (PLS): measured as the most coronal distance between the alveolar crest and the tooth surface, and III) angle between the lines (alveolar crests mesial and distal) and (cemento-enamel junction mesial and distal).

**Results:**

The mean duration of the orthodontic treatment in the test group was 2.5 years. The periodontal ligament space was statistically significantly wider on mesial areas of right molars (mean 0.2 mm, *p*<0.01), but there was no statistically significant difference found in the three other areas (distal part of the right molar, mesial and distal parts of the left molar). There was a statistically significant mean alveolar bone loss in the right and left mesial areas, respectively accounting for 0.3 mm (*p*<0.001) and 0.2 mm (*p*<0.01). No statistically significant alveolar bone loss was measured on the distal surfaces of the upper molars. The angle was wider on both sides for the test group (right *p*<0.001 and left *p*<0.05).

**Conclusions:**

A significant alveolar bone loss on the mesial tooth surface of upper first molars after orthodontic treatment was found with concurrent different levelling angles in the test group. On all other sites, no statistically significant changes were found. There was some minimal statistical significant alveolar bone loss after finishing treatment in patients who had orthodontic bands placed on their maxillary 1^st^ molars, but no clinical significance was found.

## 1 INTRODUCTION

The terminal attachments of fixed orthodontic appliances are placed on molar teeth, usually on first permanent molars. These attachments can be a cemented band or a bonded molar tube. The latter have, however, higher failure rates than molar bands [1-3], causing emergency visits, lengthening of the treatment or patient dissatisfaction. First permanent molars with bonded tubes also experience more demineralization. Because of lower failure rates and higher reliability, many orthodontists tend to favor molar bands.

It is well accepted that patients with fixed orthodontic appliances face a difficult oral hygiene situation because brackets and wires can impede oral hygiene [4]. It has been hypothesized that permanent loss of crestal alveolar bone may be the consequence of both increased retention of microbial plaque around fixed appliances and higher osteoclastic activity during tooth movement [5].

To assess changes in alveolar bone levels, radiographic evaluation is a well-accepted technique. Available information on the effect of orthodontic treatment on crestal alveolar bone levels measured radiographically is contradictory. While some studies reported no effect [6-8], others revealed more bone loss in orthodontic patients than in untreated age-matched subjects. Significant attachment loss has been reported for distal [9-11] as well as for mesial [12] surfaces of first maxillary molars. Nevertheless, these differences were small (0.2 - 0.5 mm).

Almost 20 years ago Bondemark [12] longitudinally followed two groups of 20 adolescents, one orthodontically treated and one untreated, for 5 years. The interdental alveolar bone level was estimated on bitewing radiographs. The patients in the test group were treated with repelling rare earth magnets on one side and super elastic nickel-titanium open coils on the other. Then, fixed straight-wire appliances were used in both arches. The orthodontic treatment protocol at the University of Zurich rarely involves magnets or super elastic coils to gain space in the maxilla.

However the basic Bondemark study design was reemployed, though with a larger number of patients in both the test and control groups, for this retrospective cross-sectional radiographic study to compare the alveolar bone level of banded upper first molars on bitewing radiographs taken after debonding to the bitewing radiographs of untreated controls. We expected most changes to be found at this point in time. In addition, potential changes in dimensions of the periodontal ligament space and the angulation between the cementum-enamel-junction and alveolar bone were investigated. We hypothesized that these surrogate parameters would affect the test teeth more than the control teeth *i.e.* that banded molars would show greater distances between the cemento-enamel-junction and alveolar crest, wider PDL spaces and increased angulations as untreated controls.

## 2 MATERIALS AND METHODS

This study had a retrospective cross-sectional design and assessed the alveolar bone level of upper first molars in bitewing radiographs. The study compared an orthodontic test group with an untreated control group, at one selected time point. Because bitewing and periapical radiographic techniques have provided significantly different values when assessing crestal alveolar bone levels, only bitewing radiographs were used in this study [13].

The radiographs were matched according to age and sex, to make the groups comparable. Both groups were part of the Swiss School Dental Program, were seen by a dentist on a yearly basis and had received restorative therapy or dental hygiene care when necessary. No new or additional radiographs had to be taken for this survey. The parents or caregivers had given informed consent and agreed on their child’s data being published anonymously. After anonymization any link between individuals and radiographs was no longer possible.

**Fig. (1a and b):**
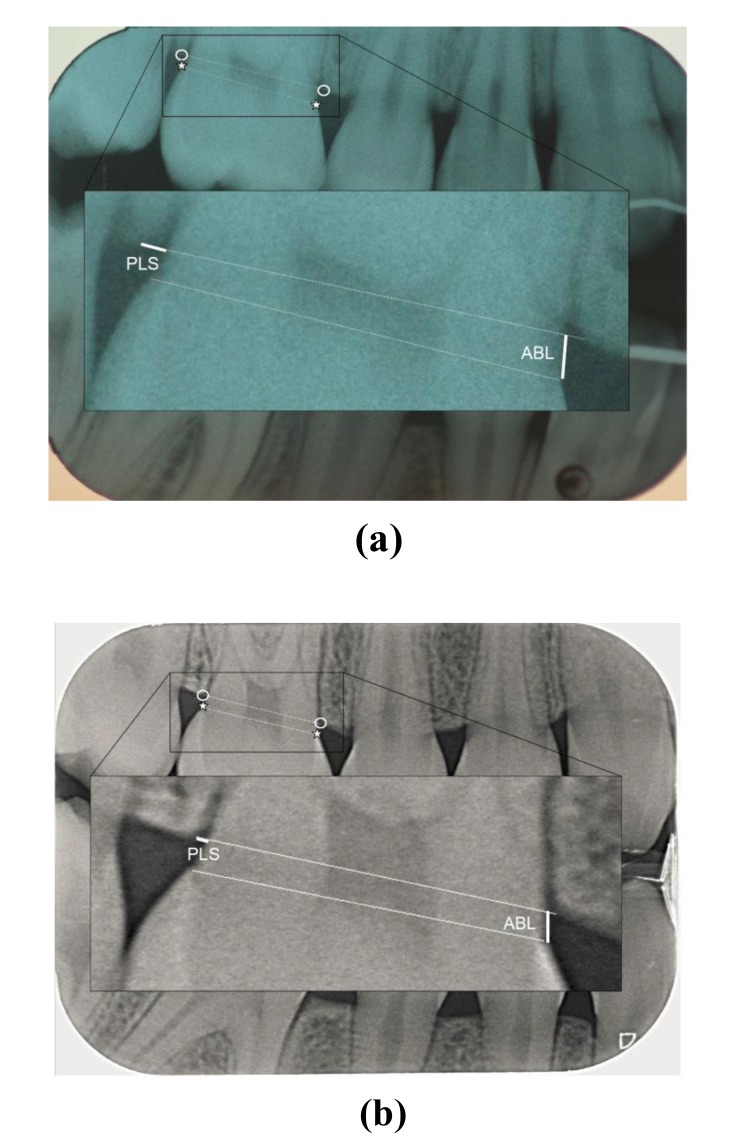
The alveolar bone level (ABL) was measured as distance between the cemento-enamel junction (CEJ, *) and a drawn line connecting the most coronal point of the interdental alveolar crest (CP, °). The periodontal ligament space (PLS) was measured as the shortest distance between the tooth surface and the respective CP, along a line connecting the mesial and distal CP. Fig. (**1a**) test group, Fig. (**1b**) control group.

**Fig. (2a and b):**
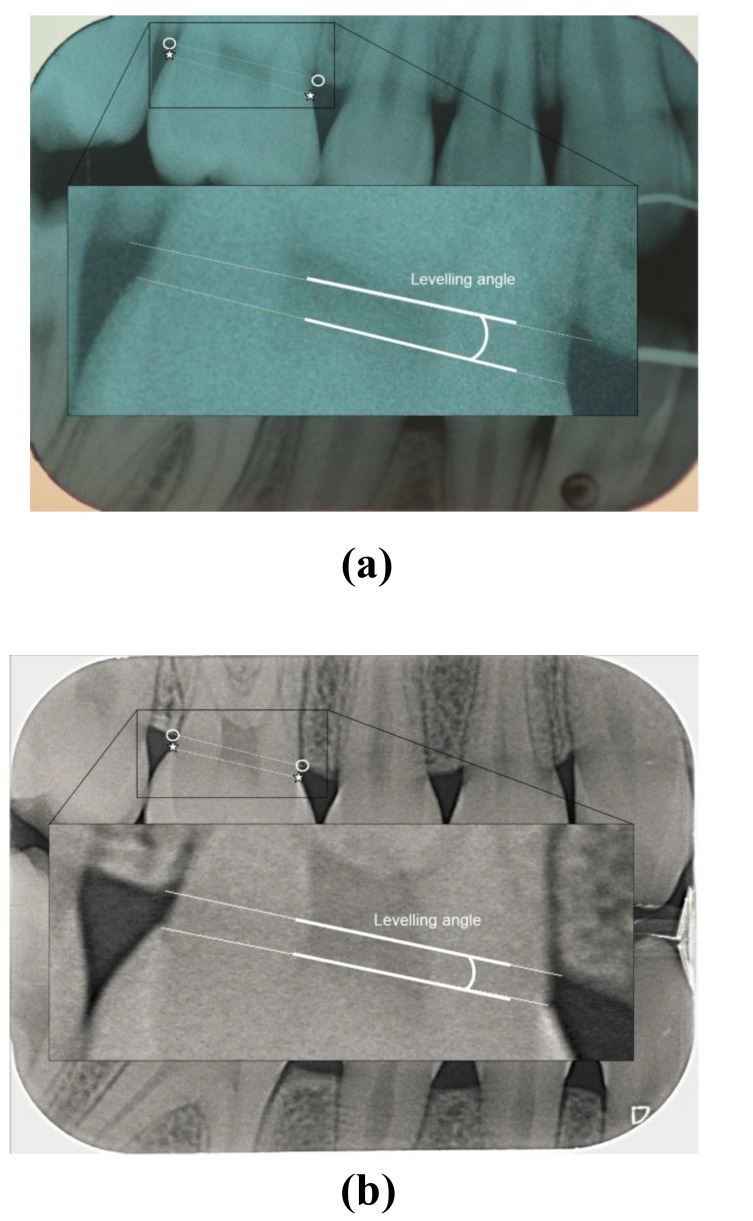
The leveling angle was measured as the angle between the line connecting the mesial and distal cemento-enamel junction (CEJ, *) and the line connecting the mesial and distal most coronal point of the interdental alveolar crest (CP, °). Fig. (**2a**) test group, Fig. (**2b**) control group.

The radiographs in Figs. (**1** and **2**) were taken from the test (Figs. **1a** and **2a**) and control group (Figs. **1b** and **2b**).

A declaration of no objection was granted by the Zurich Ethics Review Committee (Req-2016-00463).

### 2.1 Test Group

The test group consisted of subjects that had been part of an earlier investigation [14]. Bitewing radiographs were consecutively selected by one author (S.B) from the records archive in the Clinic for Orthodontics. The study inclusion criteria were: no missing or supernumerary teeth in both jaws, treatment beginning after the year 2000 to create a homogenous sample, fixed multibracket appliances in upper and lower jaw, banded first molars in the maxilla, no maxillofacial surgery, patients’ age at the end of active treatment 15.0 to 16.7 years, bitewing radiographs of high quality at the end of active treatment (debonding) showing mesial and distal aspects of upper first molars.

Forty-eight females (56%) and 38 males (44%) were included in the study. Average age at the time of bracket bonding was 13.67 years (SD ± 0.69). Average age at the end was 15.47 years (SD ± 0.35). Treatment without extractions was performed in 60 patients (69.8%), whereas four premolars were extracted in 26 patients (20.2%) for orthodontic reasons. The groups were not matched according to spacing, crowding or extraction.

Patients had been treated with fixed edgewise appliances by postgraduate students in the Clinic for Orthodontics, University of Zürich, Switzerland. The students used either a standard edgewise appliance or a pre-adjusted edgewise appliance and both used mesh-based stainless-steel orthodontic brackets with 0.018 × 0.025- in attachment slots. All patients were given a fluoride mouthwash, a toothbrush, plaque-revealing tablets and fluoride toothpaste at the beginning of fixed orthodontic treatment. Oral hygiene instructions were also given but no specific advice on interdental hygiene cleaning was provided. No restorative fillings were done during orthodontic treatment. At the day of debonding, final records including bitewing radiographs were made. The subjects’ average age at the end of treatment was 15.6 years (min 15.0, max 16.7 years) and mean treatment time was 2.5 years.

All radiographs in the test group were taken between 2002 and 2010 under standardized conditions. A Kwik-Bite film holder (Indusbello, Londrina, Brazil) and Kodak INSIGHT films were used (Speed F, 30.5 × 40.5 mm, Carestream Health, Rochester, NY, USA). The radiographs were then digitized and anonymized. Table **1** provides information regarding the study recruitment and setting for both groups.

### 2.2 Control Group

The radiographic data of another 86 individuals, 35 females (40%) and 51 males (60%), also between 14.7 and 16.7 (mean 15.5) years of age, were selected randomly from a Swiss private practice, showing mesial and distal aspects of upper first molars. These subjects had never undergone orthodontic treatment. The control group’s radiographs were taken between 2009 and 2014 under standardized conditions using a Kwik-Bite film holder (KerrHawe SA, Bioggio, Schweiz) and Dürr image plates 2+, scanned by a VistaScan Perio Plus image plate scanner (both Dürr Dental AG, Bietigheim-Bissingen, Germany). Before measuring, the radiographs were anonymized.

### 2.3 Radiographic Measurements

Radiographs were analyzed using NIH ImageJ software (National Institute of Health, USA). After calibration with a second investigator (P.S.), all radiographs were measured using *ImageJ* by one investigator (S.T.) within a 4 week time period. For the calibration process, 50 radiographs were chosen, measured by both investigators mentioned above and re-measured until a maximal deviation of less than 10% was achieved. The measurements’ reliability was assessed. Test-Retest Reliability was good (0.89).

The assessment of bone levels by direct measurement of the alveolar crest distance from a fixed reference point, such as the cemento-enamel junction was shown to be a useful diagnostic criterion for the measurement of chronic periodontitis [15] and the method of choice for valid comparisons [9]. Thus, in our study, the Cemento-Enamel Junction (CEJ) on the mesial and distal side of the upper first molar was chosen as a fixed reference point. Further the most coronal point of the interdental alveolar crest (CP) was identified mesially and distally of the first upper molar [16].

Modifying the measuring method of Zachrisson and Alnaes [10] and Janson *et al*. [17], a line through the mesial and distal Crestal Points (CP) was drawn in the program *ImageJ* at a scale of 150%. All measurements were taken at a scale of 300%. A site was scored unreadable if at least one of the reference points needed could not be identified [18].

Three parameters were measured on the digital bitewings:

- The Alveolar Bone Level (ABL) was assessed as the distance between the CEJ and a line connecting the most coronal point of the interdental alveolar crest (CP), measured mesially and distally along the tooth surface in both quadrants (Fig. **1**).
- The Periodontal Ligament Space (PLS) was measured as the shortest distance between the mesial and distal side of the tooth surface and the respective most coronal point of the interdental alveolar crest (CP), along a line connecting the mesial and distal CP (Fig. **1**).
- The levelling angle was defined as the angle between the line connecting the mesial and distal CEJ and the line connecting the mesial and distal CP (Fig. **2**).

### 2.4 Statistical Analysis

Descriptive statistics, Mann-Whitney-U, unpaired t-tests and Bonferroni correction were performed. The level of significance was set at *p* < 0.05.

## 3 RESULTS

2064 sites were measured, of which 13 sites in the control group (1.3%) and 31 sites in the test group (3.0%) could not be identified (total 2.1%).

### 3.1 Alveolar Bone Level (ABL)

Alveolar bone level measurements showed negative values for tooth surfaces where the CEJ was situated further apically than the alveolar crest. In the test group, the alveolar bone level mean values ranged between 0.56 mm to 0.92 mm. In the control group, alveolar level mean values ranged between 0.62 mm to 0.73 mm.

Unpaired t-test and Bonferroni correction for the four sites showed a significant alveolar bone loss in the test group for the mesial tooth surface on both the left and the right side (*p* < 0.01). For the distal surfaces, no significant difference was found (Table **2**).

### 3.2 Periodontal Ligament Space (PLS)

Results of the Periodontal Ligament Space (PLS) measurements are provided in Table **3**. On the upper right molar, mesial and distal aspects of the test and control teeth had mean values of 0.48 mm, 0.41 mm, 0.29 mm and 0.28 mm, respectively. On the upper left molar, mesial and distal aspects of the test and control teeth had mean values of 0.33 mm, 0.28 mm, 0.46 mm and 0.32 mm, respectively.

The four sites showed a significant difference for the mesial PLS of the right first upper molar (*p* < 0.05) but not for the remaining sites (mesial aspect of upper left first molar and distal aspects on both sides) as determined by the Mann-Whitney-U test and Bonferroni correction (Table **3**).

### 3.3 Leveling Angle

An angulation of 0° indicates a parallel orientation of the two lines measured *i.e.* the line connecting the crestal points from the mesial to the distal tooth surface and the line from mesial to distal CEJ, indicating that both mesial and distal tooth surfaces had lost no or the same amount of alveolar bone in relation to the CEJ. Such 0° angles were measured for both the test and the control group.

For teeth with negative alveolar bone levels on one side (CEJ below CP) and positive values on the other side, the two lines crossed within the tooth. These angle values were included and were observed equally in both groups.

Mean angles of 3.37° were measured for the test group on the right side and 2.31° for the control group. On the left side, angulation of 3.37° for the test group and 2.36° for the control group was measured (Table **4**).

Mann-Whitney-U test for both sides proved a significant difference between the test and the control group.

## 4 DISCUSSION

This study examined the alveolar bone level and periodontal ligament space of upper first molars in an orthodontic test group by comparing attachment levels, periodontal ligament space and leveling angles, as measured on bitewing radiographs, to an untreated control group.

In our study, a significant loss of alveolar bone was shown for mesial tooth surfaces on the left and right side in the test group, supporting earlier findings [5]. In correlation, the test group showed a significant difference of the leveling angle for both sides. Interestingly, a significantly wider dimension of the periodontal ligament space was shown only at the mesial tooth surface of the right upper first molar in the test group.

The only study known to the authors using a similar approach is one by Bondemark [12]. In contrast to this study, repelling rare earth magnets on one side and super elastic nickel-titanium open coils on the contralateral side were used to gain space in the maxillary arch. Whereas at the start of the study no statistically significant difference in the attachment level between the test and control group was observed, at the end of treatment (2.8 years) the mesial surfaces of first and second maxillary molars of the test group showed significantly larger attachment loss which was preserved at the final reading after 5 years (2 years after debonding). No values ≥ 2.0 mm between the CEJ and the alveolar crest were measured at interdental sites, which corroborates the finding that the majority of the orthodontically treated teeth showed no or little damage to the attachment apparatus [12].

In our study, mean treatment time was 2.5 years with a maximum measurement of CEJ-CP distance of 1.99 mm in the test group. The treatment time in the test group was longer compared to other university settings. According to the results of a recent systematic review, comprehensive orthodontic treatment on average requires less than 2 years to complete. But a wide range of treatment durations (14-33 months) were reported [19]. In the Zurich orthodontic postgraduate program several instructors are involved. All of them were trained at University of Zurich. Although there are always certain differences in orthodontic care, it can be assumed that there was a consistency in the orthodontic care in the postgraduate program. The study by Bondemark only comprised of 20 subjects in both groups, whereas in the present study 86 individuals per group could be included. In the treatment protocols of the Zurich postgraduate clinic, headgear is sometimes used. Forces applied in the dorsocranial direction might partially explain the fact that also in the present study, there was a significant alveolar bone loss found on the mesial sites of upper first molars.

In a study by Boyd [18], loss of attachment between the pretreatment and posttreatment examinations was significantly greater for maxillary banded molars than bonded molars. Also, banded molars had significantly more plaque accumulation and gingival inflammation than bonded molars. Diedrich *et al*. have shown that the fit of orthodontic bands significantly varies in width between the band and tooth surface in the occlusal and cervical margins compared to the equatorial area [5]. The cervical margins of the bands were located supragingivally in the buccolingual areas and subgingivally in virtually all approximal regions [5]. At the gingival margins of the bands in the interdental area, adherent plaque on the outer band surface was seen side by side with subgingival adherent and loosely dispersed plaque in cement porosities and fissures. The interdental gingiva of all banded teeth presented the histopathologic picture of an established gingival lesion [5]. Especially in the interdental region, subgingival extension of the bands with plaque-infected overhanging margins and cement defects had resulted in inflammatory destruction of the papillary architecture, of the epithelial attachment, and the intra-papillary and dentoalveolar fiber structures due to microbial invasion and accompanying specific/unspecific immunologic defense mechanisms [5]. In various studies it was concluded that orthodontic and occlusal loading might contribute to periodontal destruction in periodontally diseased patients through downregulation of matrix and osteogenic proteins and up-regulation of pro-inflammatory cytokines [20, 21]. In horizontal tooth displacement, the lowest tensile stress in the cervical region was observed in the model of normal bone height with widened PDL and an increase of maximum tensile stress was observed as the alveolar height was reduced [22, 23]. So there are several reasons for the observed periodontal reactions: mechanical irritation due to subgingival band extension and consequently subgingival plaque accumulation or cytotoxic effects of cement and/or band material. Differences in the present results between the mesial and distal sites of banded molars might also be correlated with their anatomy, showing a concavity on the mesial side [24].

As the measurements are of very small dimension, the calibration process proved to be rather elaborate. Defining the points of measurements was demanding even at a large scale of 150% and 300% respectively.

The measurements were taken by one investigator after calibration. In order to minimize errors and enhance repeatability all measurements were taken within a short period of time. Further limitations of this retrospective study include missing observations before orthodontic treatment. As this study was a radiographic one there were no clinical parameters available. Of courses this would have been helpful and could be included in further studies.

Another shortcoming of the study is that there were no standard initial radiographs in both groups. The pretreatment radiographs available in the orthodontic test group were taken at various ages (9-14years) and thus were not comparable. According to Swiss radiographic guidelines bitewing radiographs should not be taken before the age of 14 in patients without carious lesions [25]. The private practice that provided the control group’s radiographs also follows these guidelines. Therefore, bitewing radiographs for the control group were not available unless a patient hat experienced a carious lesion prior to his/her 15^th^ birthday.

In the test group digital bitewing radiographs were used whereas in the control group analog ones were used. Although this might present a method error, a study reported no significant differences in the diagnostic accuracy of analog (Insight film) and digital (Digora) radiographs [26].

A prospective study could not be carried out for of ethical reasons, especially in light of radiation exposure in adolescents. Although there are some limitations and shortcomings in this retrospective study, it excels by comparing two rather large age-matched groups showing similar characteristics. The only difference was having undergone orthodontic treatment or not. The measurements were taken at debonding and temporary adaptation of the periodontal ligament frequently lead to increased mobility after orthodontic movements. This normally is self-correcting after a short period of stabilization and occlusal adjustments.

In summary, it was possible to examine two large, comparable samples with and without orthodontic treatment. The subjects in both groups lived in the same area and had a comparable socioeconomic status. The measured changes in the bitewing radiographs were very small and not clinically significant in any parameter. The clinical implications include accurate planning of force distribution during orthodontic therapy as well as consideration of regular dental hygiene treatment. More scientific research will be needed to define models suitable to investigate biomechanics of tooth movements and their implications on the periodontal ligament complex.

## CONCLUSION

In our retrospective cross-sectional radiographic study a significant alveolar bone loss on the mesial tooth surface of upper first molars after orthodontic treatment was found with concurrent different levelling angles in the test group.

On all other sites, no statistically significant changes were found. There was some minimal statistical significant alveolar bone loss after finishing treatment in patients who had orthodontic bands placed on their maxillary 1^st^ molars, but no clinical significance was found.

## Figures and Tables

**Table 1:** Patient characteristics and details of the study recruitment.

–	Test	Control
Number (male/female)	86 (48/38)	86 (51/35)
Average age (range)	15.6 (15.0 – 16.7)	15.5 (14.7 – 16.7)
Orthodontic treatment	yes	no
Bitewing radiographs	Analog, Kodak Insight films, Speed F	Digital, Dürr image plates 2+ & Dürr VistaScan Perio Plus image plate scanner
Period	2002 – 2010, (day of debonding)	2009 – 2014 (routine examinations)
Origin of radiographs	University	Private Practice
Anonymization	yes	yes
Percentage of sites not identified	3.0%	1.3%

**Table 2:** Mean alveolar bone levels (measured in mm, confidence intervals in brackets) of the radiographic measurements of the left and the right first molars at the mesial and distal aspects of test and control teeth and pairwise comparison.

–	Right Quadrant		Left Quadrant	
	Mesial	Distal	Mesial	Distal
Test	0.92 (-0.77 - 1.99)	0.60 (-2.12 - 1.65)	0.92 (-0.15 - 2.08)	0.56 (-086 - 2.56)
Control	0.62 (-0.40 - 2.40)	0.73 (-0.90 - 2.01)	0.73 (-0.39 - 1.91)	0.62 (-1.55 - 1.56)
*p*-value	*p<0.01*	0.1018 (n.s.)	* p<0.01*	0.4879 (n.s.)

**Table 3:** Periodontal ligament space (measured in mm, confidence intervals in brackets) of the radiographic measurements of the left and the right first molars at the mesial and distal aspects of test and control teeth and pairwise comparison.

–	Right Quadrant		Left Quadrant	
	Mesial	Distal	Mesial	Distal
Test	0.48 (0.07 - 2.13)	0.41 (0.07 - 1.60)	0.32 (0.05 - 1.41)	0.46 (0.05 - 2.03)
Control	0.29 (0.04 - 1.12)	0.28 (0.06 - 0.82)	0.28 (0.06 - 0.78)	0.32 (0.07 - 1.02)
*p*-value	*p<0.05*	0.0839 (n.s.)	0.8675 (n.s.)	0.3232 (n.s.)

**Table 4:** Mean levelling angle measurements [°]. Confidence intervals in brackets.

–	Right Levelling Angle	Left Levelling Angle
Test	3.37° (0°-15.0°)	3.37° (0°-12.0°)
Control	2.31° (0°-12.0°)	2.36° (0°-11.0°)
*p*-value	0.0060 (*p*<0.01)	0.0186 (*p*<0.05)

## LIST OF ABBREVIATIONS

ABL: = Alveolar Bone Level
CEJ: = Cemento-Enamel Junction
CP: = Crestal Point; most coronal point of the interdental alveolar crest
PLS: = Periodontal Ligament Space
n.s.: = not significant
